# Interleukin 13 signaling modulates dopaminergic functions and nicotine reward in rodents

**DOI:** 10.1038/s41380-025-03137-3

**Published:** 2025-08-07

**Authors:** Xin-an Liu, Carlos A. Aguirre, Qixing Yang, Jiayan Ren, Lina Wang, Furong Ju, Hongling Guo, Jintao Wang, Luping Liu, Zixuan Li, Juan He, Zhibin Xu, Cuihan Shi, Rigo Cintron-Colon, Tatiana Michel, Malcolm Wood, Alexander V. Margetts, Tate A. Pollock, Samara J. Vilca, Luis M. Tuesta, Silvia Di Angelantonio, Bernadette Basilico, Maria Egle De Stefano, Jianxun Xia, Pengfei Wei, Shupeng Li, Xiaofei Yang, Liping Wang, Violaine D. Delorme-Walker, Maria Cecillia Garibaldi Marcondes, Loren Parsons, Bruno Conti, Zuxin Chen

**Affiliations:** 1 Guangdong Provincial Key Laboratory of Brain Connectome and Behavior, the Brain Cognition and Brain Disease Institute, Shenzhen-Hong Kong Institute of Brain Science, Shenzhen Institutes of Advanced Technology, Chinese Academy of Sciences, Shenzhen, China; 2 University of Chinese Academy of Sciences, Beijing, China; 3 Nash Family Department of Neuroscience, Icahn School of Medicine at Mount Sinai, New York, NY USA; 4 Scripps Research; 10550 North Torrey Pines Road, La Jolla, CA USA; 5 Shenzhen Key Laboratory of Drug Addiction, Shenzhen Neher Neural Plasticity Laboratory, Shenzhen-Hong Kong Institute of Brain Science, Shenzhen Institutes of Advanced Technology, Chinese Academy of Sciences, Shenzhen, China; 6 Key Laboratory of Cognitive Science, Hubei Key Laboratory of Medical Information Analysis and Tumor Diagnosis & Treatment, Laboratory of Membrane Ion Channels and Medicine, College of Biomedical Engineering, South-Central Minzu University, Wuhan, China; 7 State Key Laboratory of Oncogenomics, School of Chemical Biology and Biotechnology, Peking University Shenzhen Graduate School, Shenzhen, China; 8 Luxembourg Institute of Health, Strassen, Luxembourg; 9 Department of Psychiatry and Behavioral Sciences, University of Miami Miller School of Medicine, Miami, FL USA; 10 Department of Physiology and Pharmacology, Sapienza University, Rome, Italy; 11 Center for Life Nano- & Neuro-Science of Istituto Italiano di Tecnologia (IIT), Rome, Italy; 12 Department of Biology and Biotechnologies “Charles Darwin” and Center for Research in Neurobiology “Daniel Bovet”, Sapienza University of Rome, Rome, Italy; 13 Yunkang School of Medicine and Health, Nanfang College, Guangzhou, Guangdong China; 14 San Diego Biomedical Research Institute, San Diego, CA USA

**Keywords:** Addiction, Neuroscience

## Abstract

Neuroimmune signals can regulate neuronal function and affect behavior through mechanisms that are not yet fully understood. Here we investigated the action of interleukin 13 (IL-13), a cytokine that can be produced in the brain by both microglia and neurons. We show that dopamine-containing neurons in the ventral tegmental area (VTA) predominantly express the IL-13 receptor alpha 1 (IL-13Rα1) and exhibit presynaptic vesicular localization of neuronal IL-13. Exogenous application of IL-13, or its endogenous mobilization by optogenetics, reduced the activity of VTA dopaminergic neurons and opposed the stimulatory effects of nicotine on these neurons in rodents. These actions required IL-13Rα1, activation of the PI3K/AKT pathway, and functional hyperpolarization-activated cyclic nucleotide-gated (HCN) channels. Consistently, local infusion of IL-13 into the VTA markedly reduced nicotine self-administration in rodents. Collectively, these findings demonstrate that IL-13 acts in a neuromodulator-like fashion on mesolimbic dopamine neurons expressing IL-13Rα1. Our data also indicate that IL-13Rα1 signaling regulates the stimulatory actions of nicotine, suggesting a potential role for this neuronal immune signaling in reward processing and the addictive properties of nicotine.

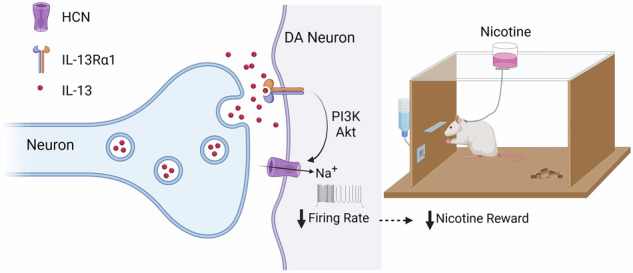

## Introduction

Neuroimmune signals are increasingly recognized as modulators of neuronal function and complex behavior, including addiction [1–6]. Among them are cytokines, small proteins that act as signaling molecules in autocrine, paracrine, or endocrine fashion. Interleukins are cytokines, originally identified as leukocytic factors, inducible during infection or tissue damage to regulate peripheral immune responses by promoting or reducing inflammation [7]. These proteins are also considered to be mediators of sickness behavior, a motivational state commonly observed during infection and charaterized by lethargy, loss of appetite, malaise, depression, and anhedonia [8–10]. Peripheral cytokines are considered too large to readily and freely cross the intact blood brain barrier, although, for some, regulated transport has been described [11]. In addition, work from several laboratories, including our own, demonstrated that some cytokines can be produced in the brain [4, 12, 13]. Among these is interleukin-13 (IL-13) [14–17].

IL-13 is a pivotal modulator of peripheral allergic asthma and helminthic responses, produced by several cell types including mast cells, basophils, NK cells, and CD4^+^ T lymphocytes [18]. Two receptors for IL-13 have been identified, both forming heterodimers with the interleukin-4 receptor alpha (IL-4Rα). One is the canonical IL-13 receptor alpha type 1 (IL-13Rα1), which activates the JAK/STAT and/or the PI3K/AKT/mTOR pathways [19–21]. The other is IL-13Rα2, a protein lacking an intracellular domain and therefore generally regarded as a decoy negative regulator of IL-13 action, although it has been reported to participate in the regulation of TGF-β1 signaling [22]. Since the 13Rα1/IL-4Rα receptor can be activated by IL-13 as well as by IL-4, these two cytokines are often investigated in parallel. However, because IL-4 also binds to a second independent receptor, IL-4Rα/IL-2rg, IL-13 and IL-4 do not necessarily act in a redundant manner. In the brain, IL-13 is found in the cerebrospinal fluid and can be produced by microglia during neuroinflammation as well as by neurons, whereas the likely source of IL-4 in the CNS has been mainly attributed to infiltrating T cells [14, 15, 17, 23–25]. Expression of IL-13Rα1 is high in midbrain dopaminergic (DA) neurons, is inducible in noradrenergic cells of the locus coeruleus neurons, and has also been reported in cortical neurons and activated microglia [15, 16, 26, 27]. Conversely, IL-13Rα2 is expressed in most glioblastoma but not in the healthy brain [28].

The current knowledge of IL-13 and IL-4 in the CNS derives mainly from studies investigating the role of neuroinflammation in neurodegeneration in pathological models. Both cytokines are classified as anti-inflammatory for their ability to promote a Th_2_ response and to downregulate the expression of several pro-inflammatory molecules [24, 29–34]. Data to date indicate that IL-13 and IL-4 can contribute to both neuroprotection and neurodegeneration depending on the experimental model and the neuronal population investigated. Administration of either peripheral or central exogenous IL-13 conferred protection in experimental models of traumatic brain injury and ischemia [17, 35, 36]. Neuroprotection was also observed with IL-4 in models of intracerebral hemorrhage, ischemia and spinal-cord injury [37–40]. These actions have primarily been attributed to the anti-inflammatory properties of IL-13 and IL-4, and may also be explained in part by their ability to reduce microglia activation by increasing cellular susceptibility to oxidative-stress-mediated damage [26, 27]. Intriguingly, we found that exogenous IL-13 or IL-4 also increased the vulnerability of dopaminergic neurons expressing IL-13Rα1/IL-4Rα to oxidative damage, thus potentially contributing to neurodegeneration [14–16, 41, 42]. Despite this, we also showed that IL-13 and IL-4 are not intrinsically toxic and do not damage dopaminergic cells in the absence of a sufficient level of free radicals [14–16].

Here, we begin to characterize the physiological roles that IL-13 and IL-4 may have in the brain. We focused on VTA DA neurons expressing IL-13Rα1/IL4Rα and examined the electrophysiological effects of IL-13 on these cells. We also assessed whether IL-13Rα1 regulated nicotine consumption. In fact, nicotine exerts stimulatory effects on VTA DA neurons that are considered both necessary and sufficient for the reinforcing properties of the drug that supports the development of tobacco addiction [43, 44]. In addition, studies have found that polymorphisms in IL-13, the endogenous ligand of IL-13Rα1, have been associated with an increased risk of smoking-related lung diseases, possibly due to higher cigarette consumption in affected individuals [45–50].

## Methods

### Animals

Male adult C57BL/6J mice were purchased from GemPharmatech (Nanjing, China) or from Charles River (Calco, Italy). IL-13Rα1 knock-out (IL-13Rα1 KO) mice used for breeding were purchased from Shanghai Model Organizations (Cat# NM-KO-190457l; Shanghai, China). IL-13Rα1 KO mice were then crossed with C57BL/6J mice, the offspring male IL-13Rα1 KO mice and their WT littermates were used for experiments. DAT-Cre mice were purchased from The Jackson Laboratory (Jax #006660). All the mice were housed in groups of 5 per cage at 22–25 °C and 30–40% humidity on a 12-h light/dark cycle with food and water available *ad libitum*. Male Wistar rats weighing 300–320 g were purchased from Charles River and housed in groups of two per cage in a controlled vivarium with *ad libitum* food and water until nicotine self-administration training began. During behavioral testing, rats were mildly food restricted to approximately 90% of their free-feeding body weight, while water remained available *ad libitum*. All animal care and experimental procedures were conducted in strict accordance with institutional and international ethical guidelines. These include protocols approved by the Institutional Animal Care and Use Committees (IACUC) of Scripps Research, the Icahn School of Medicine at Mount Sinai, and the Chinese Academy of Sciences Shenzhen Institutes of Advanced Technology. Experiments conducted in Italy complied with national and European regulations, as approved by the Italian Ministry of Health (Authorization No. 253/2015-PR) and in accordance with the European Communities Council Directive 2010/63/EU on the protection of animals used for scientific purposes. All efforts were made to minimize animal suffering and reduce the number of animals used.

### Drugs

Recombinant mouse IL-13 (Cat# 413-ML), IL-4 (Cat# 404-ML), IL-2 (Cat# 402-ML) were purchased from R&D Systems. These cytokines were reconstituted in sterile PBS and diluted to the appropriate experimental concentrations in artificial cerebrospinal fluid (aCSF) for calcium imaging and electrophysiological recordings. PI-103 (Cat# A3724) was purchased from APExBIO, AKT inhibitor III (Cat# abs810176) from Absin, and ZD7288 (Cat# 1000) from Tocris Bioscience. Recombinant rat IL-13 (Cat# 1945-RL; R&D Systems) was reconstituted at 10 μg/mL in sterile PBS and further diluted in sterile saline to working concentrations for food and nicotine self-administration experiments. Sterile saline served as the vehicle control. (−)-Nicotine tartrate (Sigma-Aldrich, Natick, MA) was dissolved in 0.9% sterile saline, and the pH was adjusted to 7.4 with NaOH.

### Brain slices preparation

Acute midbrain slices were prepared from 3-month-old C57BL/6J male mice as previously described [51, 52]. Animals were decapitated under halothane anesthesia, and whole brains were rapidly extracted and immersed in ice-cold cutting solution continuously oxygenated with 95% O_2_ and 5% CO_2_ and containing (in mM): KCl 2.5, CaCl_2_ 2.4, MgCl_2_ 1.2, NaHPO_4_ 1.2, glucose 11, NaHCO_3_ 26, and glycerol 250 (300–310 mOsm, 7.2–7.4 pH). Horizontal brain slices (250 μm thick) containing the VTA were prepared in the ice-cold cutting solution at 4 °C using a vibratome (DSK, Kyoto, Japan). Slices were allowed to recover for at least 1 h at room temperature prior recordings in oxygenated aCSF containing (in mM): NaCl 125, KCl 2.5, CaCl_2_ 2, MgCl_2_ 1, NaHPO_4_ 1.2, NaHCO_3_ 26, and glucose 10 (300–310 mOsm, 7.2–7.4 pH). All recordings were performed at 33 ± 1 °C on slices submerged in aCSF and perfused (1 mL/min) with the same solution in the recording chamber under the microscope [53–57].

### Electrophysiology recordings

Experiments were carried out as previously described [51, 52]. Patch-clamp recordings in whole-cell mode were obtained with glass electrodes (3.7–4 MΩ) filled with (in mM): K-gluconate 145; MgCl_2_ 2; CaCl_2_ 0.1; EGTA 0.75; HEPES 10; MgATP 2 mM; Na3GTP 0.3 (290–295 mOsm, pH 7.3). Putative dopamine neurons were identified using well-established criteria, including spontaneous single-pacemaker firing at 1–4 Hz and the presence of a hyperpolarization-activated inward current (*I*_*h*_) in response to voltage steps to - 120 mV from a holding potential of −60 mV. To further confirm the neuronal identity, biocytin (0.4%) was always added to the internal solution to label the recorded neurons. Only neurons double-labeled with biocytin and TH^+^ (see immunohistochemistry section) were included in the analysis. Input resistance was monitored using hyperpolarizing pulses while holding the cell in current-clamp mode. To evaluate the effect of IL-13 on spontaneous firing rate, IL-13 (10 ng/mL) was dissolved in the aCSF. After the establishment of a stable baseline (10–15 min), IL-13 was applied for 15 min and then washed out. For the pretreatment experiments, ZD7288 (10 μM), or PI-103 (1 μM), or AKT inhibitor III (1 μM) was applied in the recording solution for 20–30 min before the whole-cell recordings. After the establishment of a stable firing rate under the pretreatment (5–10 min), IL-13 was applied as described above. The mean firing frequency (in Hz) of each neuron was calculated over the period during which cell firing stabilized (usually 4–6 min). Changes in membrane potential in spontaneously active DA cells were assessed by measuring membrane potential values between spikes. For *I*_*h*_ activation curves, hyperpolarizing voltage steps from −60 mV to −130 mV in 10 mV steps were applied from a holding potential of −60 mV. Tail currents were measured at −130 mV. Tetraethylammonium chloride (TEA-Cl, 10 mM) was added to block voltage-dependent potassium channels. Tail current amplitudes were plotted as a function of the test potentials after subtraction of the current following no hyperpolarizing step. The *I*_*h*_ activation curve was fitted with a Boltzmann function *I* = *I*max/exp[(*V* – *V*1/2)/s], where *I*max is the maximal tail current amplitude, *V* is the test potential, *V*1/2 is the half-activation potential, and *s* is the slope factor. Signals were filtered at 1 kHz, digitized at 10 kHz and acquired using an Axopatch 200B amplifier (Axon Instruments) and a Digidata 1440 A system. Recorded signals were analyzed offline using Clampfit 10 software (Molecular Devices).

### Cannula implantation and intracerebral injection procedure

Rats prepared with intracerebral cannulation were first anesthetized by inhalation of 1–3% isoflurane in oxygen and positioned in a stereotaxic frame (Kopf Instruments). Bilateral stainless-steel guide cannula (22-gauge, 18 mm in length) were implanted 1 mm above the VTA (antro-posterior, −5.8 mm from bregma; medio-lateral, ± 2.40 mm; dorso-ventral, −7.0 mm from skull; 10^°^ towards midline). Four stainless steel skull screws and dental acrylic held the cannula in place. IL-13 was injected bilaterally into VTA at a volume of 1.5 µL per side over 3 min. Injectors were left in place for at least 2 min following injections.

### Food and intravenous nicotine self-administration (IVSA)

For rat nicotine IVSA experiments, Wistar rats were mildly food restricted to ~90% of their *ad libitum* body weight and trained to respond to “active” lever pressing in operant chambers (Med Associates, St. Albans, VT) for food pellets (45 mg food pellets; TestDiet, Richmond, IN) under a fixed-ratio 5, time out 20 s (FR5TO20) schedule during 1-h daily sessions, following recovery from VTA cannulation surgeries. An “inactive lever” was also presented and recorded. Once stable responding was achieved ( > 90 pellets per session), rats were catheterized into the right jugular vein as previously described [58]. After at least 4 days of recovery, the rats resumed food training under the same FR5TO20 schedule. Once food-responding criteria were re-established, the animals were allowed to acquire intravenous nicotine self-administration (0.03 mg/kg/infusion) during 1-h daily session under the FR5TO20 schedule for at least 7 consecutive sessions before treatment with IL-13. As in food self-administration sessions, both active and inactive levers were presented, but only active lever pressing triggered nicotine infusions. Completion of an FR5 on the active levers resulted in an intravenous infusion of nicotine (0.03 mg/kg nicotine free base, in a volume of 0.1 mL, delivered over 1 s). For testing the effects of IL-13 on nicotine intake, stable responding for nicotine was re-established between IL-13 treatments, defined as ≤20% variation in the number of infusions earned per session over at least 2 consecutive sessions. Rats were back to food responding sections after completion of IL-13’s effect on nicotine intake. Once stable responding was achieved again, IL-13’s effect on food responding was tested. All IL-13 treatments were delivered into the VTA of rats using an ascending doses design (0, 15 ng, 45 ng), right before the testing for nicotine or food responding. The highest doses was below 60 ng commonly used to examine centrally administered cytokines [59]. To directly compare the effects of IL-13 on responding for nicotine versus food rewards, intake data for each condition were expressed as percentage change from baseline (i.e., the mean number of rewards earned after vehicle treatment).

### Immunohistochemistry

Slices used for electrophysiology were fixed immediately after recording in 4% paraformaldehyde for 24 h and then stored at 4 °C in PBS. After Triton X100 permeabilization, injected biocytin was revealed using FITC-conjugated streptavidin (5 h at RT) (Invitrogen); then sections were stained with primary antibody (overnight at 4 °C) anti-TH (Millipore,USA Cat# AB152) 1:1000, and secondary antibody conjugated to AlexaFluor594 (2 h at RT). Confocal microscopy analysis was performed with a FV1200 (Olympus) Multiphoton System, at 60x (water immersion object) magnification. Three dimensional reconstructions of TH^+^ and biocytin injected neurons were obtained from z-stacks with Surface module of Imaris software (Bitplane, Zurich, Switzerland).

Slices used for immunohistochemistry of TH and IL-13Rα1 were permeabilized in 0.3% Triton X100 + 0.5% BSA + 0.1 M Phosphate buffer. Slices were washed, blocked and incubated with primary antibodies anti-TH (ThermoFisher Scientific, USA, Cat# PA1-4679, dilution 1:1000) and anti-IL-13Rα1 (ThermoFisher Scientific, USA, Cat# PA5-50989, dilution 1:200) overnight at 4 °C. Slices were then incubated with secondary antibodies Donkey anti-Rabbit AlexaFluor647 or Donkey anti-Sheep AlexaFluor488 (ThermoFisher Scientific, USA, dilution 1:500) in combination with Hoechst (ThermoFisher Scientific, USA, Cat# H3570, dilution 1:2000) to detect nuclei (4 h at RT). Slices were mounted on slides with Prolong Gold Antifade (ThermoFisher Scientific, USA, Cat# P36934). Confocal images were acquired on a Zeiss LSM 880 laser scanning confocal using Zeiss 20x and 40x objectives. Images were analyzed using ImageJ/FIJI (NIH).

### Immuno electron microscopy

Brains were collected from mice perfused with 0.1 M PB, followed by a solution containing 4% paraformaldehyde and 0.05% glutaraldehyde in PBS. Brains were stored for 4 h before being sliced with a vibratome to obtain 200 µm thick sections that were washed overnight in PBS. Endogenous peroxidase was quenched by incubating sections in 3% dH_2_O_2_ for 15 min followed by washings in 0.1 M PB (3 × 5 min). Sections were then permeabilized for 1-h with 0.1% Tween 20 in PBS and washed in 0.1 M PB (3 × 5 min) and were incubated for 15 min in 50 mM glycine in PBS to quench free aldehydes. All these steps were performed at 4 °C. Blocking was performed for 1 h at room temperature with 5% normal horse serum plus 1% BSA in 0.1% Tween 20 in PBS followed by incubation with primary Ab goat anti-mouse IL-13 (R&D System; Cat# AF-413-NA) 1:20 in 0.5% BSA, 0.1% Tween 20 in 0.1 M PB, overnight incubation at 4 °C. The Ab was antigen affinity purified and tested for its ability to neutralize IL‑13-induced activity. This Ab failed to detect any staining in the brain of IL-13KO animals compared to those from WT mice in previous experiments performed to determine IL-13 distribution in the CNS [15]. Sections were washed in 0.5% BSA in 0.1 M PB (3 × 5 min) and incubated in secondary Ab (Donkey anti-goat, Vector Labs, 1:200 in 0.5% BSA in 0.1 M PB) for 1 h at room temperature and, after washing in 0.5% BSA in 0.1 M PB (3 × 5 min), they were incubated with Vectastain ABC reagent for 30 min at room temperature and washed in 0.1 M PB (3 × 5 min) following the manufacturer’s instruction. DAB staining was performed for 5 min in ImmPACT DAB peroxidase substrate for (1:30 ImmPACT DAB, Vector Labs reagent). After washing for 10 min at room temperature in 0.1 M Na cacodylate buffer PBS, sections were fixed in 2.5% glutaraldehyde in 0.1 M Na cacodylate buffer (pH7.3) overnight at 4 °C, washed again for 10 min 0.1 M Na cacodylate buffer and post-fixed in 1% OsO_4_ in 0.1 M Na cacodylate buffer for 1 h. Sections were then washed in 0.1 M Na cacodylate buffer (2 × 5 min) and dehydrated in 50%, 70%, 90% graded ethanol followed 100% ethanol and transitioned in propylene oxide before being placed in either 100% Epon - Araldite resin or LR White resin to impregnate overnight. The vibratome sections were flat embedded in the resin and polymerized overnight at 60°C. Ultrathin sections (60 nm) were cut on an ultratmicrotome, mounted on copper mesh grids and examined on a Philips CM100 Electron Microscope at 60 kV (Thermo Fisher Scientific, Waltham, MA). Images were documented with an Olympus-SIS Megaview III CCD camera (Lakewood, CO).

### Cell type specific RNA sequencing

The method was similar to that previously described [60]. Briefly, AAV-KASH-HA virus (AAV-pEF1a-FLEX-HA-VHH-KASH-WPRE, Addgene #129704) was injected into VTA of Dat-Cre mice. 4 weeks later, VTA containing tissues of these mice were collected for nuclear isolation and immunolabeling. Immunolabeling nuclei were then sorted based on presence of both DAPI and HA-tag using the FACS Aria II Fusion (BD) at the University of Miami Flow Cytometry Core. Total RNA from 500-1000 nuclei was extracted and normalized for input for RNA sequencing. RNAs were reverse transcribed and amplified; resulting cDNAs were then enzymatically fragmented following manufacturer’s recommendation (New England Biolabs). Single-end 100 bp sequencing was performed on a NovaSeq6000 sequencer (Illumina) at the University of Miami. All RNA-Seq data used in this study were mapped to the mm10 genome. Reads with a length of at least 20-bp were mapped to the genome using STAR (v.2.7.8a). The resulting bam files were then passed to StringTie (v.2.1.5) to assemble sequenced alignments into estimated transcript and gene count abundance given the Gencode GRCm39 (v.M22) transcriptome assembly. For differential gene expression analysis, only genes with transcript abundance > 10 across samples, showing more than a two-fold expression change and a q-value < 0.05 were considered as differentially expressed.

### Tissue lysis and Western blotting

Acute brain slices containing VTA were prepared as described in the *2.4*. section. A total of 48 acute brain slices were prepared from 24 male C57BL/6J mice (2 acute slices from each mouse). After recovery from the preparation, slices were incubated for 20 min in the aCSF with or without IL-13 (10 ng/mL), then both sides of the VTA-enriched area for each brain slice were punched out for Western blotting analysis ( ~ 4 mg/tissue) [61]. Brain tissues were mechanically homogenized in RIPA buffer (Beyotime, P0013B) and incubated on ice for 30 min. Homogenates were centrifuged at 10,000 g for 10 min at 4 °C. Supernatants were used as total protein lysates and were analyzed by SDS–PAGE. A prestained standard (GoldBand Plus 3-color Regular Range Protein Marker, Yeasen, 20350ES90) was used as a size marker for western blotting. The antibodies used for western blotting were HCN2 (Proteintech, Cat# 55245-1-AP), STAT6 (SAB Signalway Antibody, Cat# 48795), STAT3 (CST, Cat# 9139 s), p-STAT3 (CST, Cat# 9134 s), JAK2 (CST, Cat# 3230 s), ERK1/2 (Proteintech, Cat# 11257-1-AP), p-ERK (CST, Cat# 4370 s), GAPDH (Proteintech, Cat# HRP-60004) and horseradish peroxidase (HRP)-conjugated secondary antibodies (Santa Cruz Biotechnology, Cat# sc-2030).

### Statistical analysis

Data, analyzed offline, are presented as mean ± SEM. Statistical analyses were performed using Origin 7, Prism 8 (Graphpad Software) and SigmaPlot 11 (Systat Software Inc., San Jose, CA, USA). Data normality was assessed using the Shapiro-Wilk test and variance chi-square was assessed using the Levene test. If data satisfied normality and homogeneity of variance, ANOVA, paired t-tests, or independent t-tests were used, as indicated in the figure legends

## Results

### IL-13Rα1 is predominantly expressed in the VTA DA neurons

**Fig. 1:**
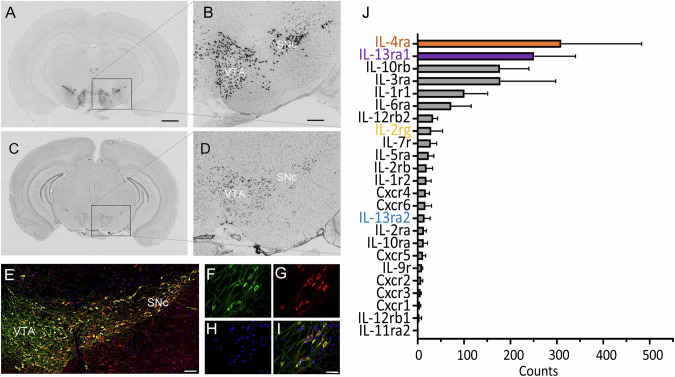
IL-13Rα1 is one of the most prevalent immune receptors in the VTA TH^+^ neurons. **A**–**D** Representative in situ hybridization images for tyrosine hydroxylase (TH) (**A**), (**B**) and IL-13Rα1 (**C**), (**D**) in coronal sections of a naïve C57BL/6J male mouse. SNc, substantia nigra pars compacta; VTA, ventral tegmental area. Scale bar: 1 mm (**A**), (**C**), 300 μm (**B**), (**D**). Image credit: Allen Institute, http://mouse.brain- map.org/. **E** Representative confocal microscopy images showing co-localization of TH (green) and IL-13Rα1 (red) in the VTA and SNc. Nuclei were stained with Hoechst (blue). Scale bar: 150 µm. **F**–**I** A representative magnified view of VTA in (**E**), showing the immunostaining of TH (**F**), IL-13Rα1 (**G**), Hoechst (**H**), and their co-localizations (**I**) in the VTA neurons. Scale bar: 60 µm. **J** Summary of mRNA levels of immune receptors in the midbrain dopaminergic neurons. Data were obtained by cell type specific nuclei RNA sequencing of mice midbrain tissues. Data are presented as mean ± SEM.

To determine the central roles of IL-13, we first investigated the distribution pattern of its receptor, IL-13Rα1, across brain regions. In the mouse brain, IL-13Rα1 expression is predominantly in midbrain dopaminergic neurons of the ventral tegmental area (VTA) and the substantia nigra pars compacta (SNc) (Fig. 1A–I), where we previously determined that approximately 80% of tyrosine hydroxylase positive (TH^+^) neurons in the VTA/SNc are also IL-13Rα^+^ [14–16]. Results obtained from TH^+^ type specific nuclei sequencing of mice midbrain tissues also showed that IL-13Rα1 and IL-4Rα, the two subunits of the canonical functional IL-13 receptor, are the most abundantly expressed immune receptors in the midbrain dopamine neurons **(**Fig. 1J**)**.

### Neuronal IL-13 localizes at the presynaptic vesicles

**Fig. 2:**
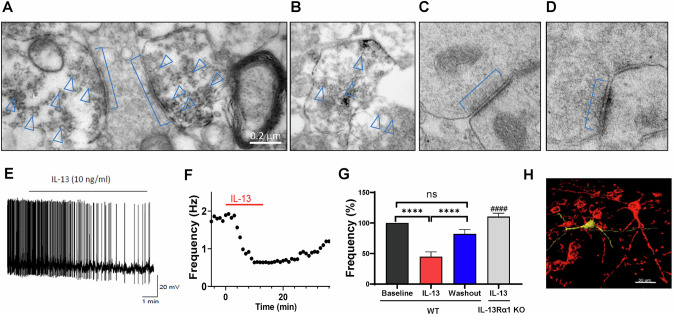
IL-13 negatively regulates the activity of VTA TH^+^ neurons. **A**–**D** Representative Immuno-EM images showing that IL-13 localized at the presynaptic vesicles adjacent to the active zone. Square brackets indicate synapses, and arrowheads indicate vesicle staining. (**A**), IL-13-positive presynaptic staining; (**B**), IL-13-positive axonal staining; (**C**), IL-13 negative presynaptic staining; (**D**), Control staining without primary anti-IL-13 antibody. **E** Sample trace showing the effect of IL-13 (10 ng/mL) on the spontaneous activity of a TH^+^ VTA neuron. **F** Time course of mean firing frequency of a VTA TH^+^ neuron at baseline, during and after IL-13 application. **G** Bar chart showing the change in firing frequency of VTA TH^+^ neurons at 15 min post-IL-13 application and 20 min post-washout (n = 18 cells). The firing frequency of each cell was normalized to its baseline level and presented as a percentage. F(2, 33) = 26.17, p < 0.0001, One-way repeated measures ANOVA, for main treatment effect; ****, p < 0.0001; ns, not significant; post-hoc Tukey’s multiple comparisons test among means. Note that, the grey bar shows the effect of IL-13 on the VTA TH^+^ neurons in IL-13Rα1 Knockout mice (n = 8 cells, from 4 mice). The suppression effect of IL-13 was abolished by deletion of IL-13Rα1. ^####^, p < 0.0001, compared to the effect of IL-13 in the C57BL/6J mice; unpaired t test. **H** Representative post-recording double immunohistochemistry performed used to demonstrate the identity of the recorded neurons stained for the injected biocytin (AlexaFluor 594; which is showed in yellow in H) and for tyrosine hydroxylase (AlexaFluor 488; which is showed in red in H). Data are presented as Mean ± SEM.

After determining the brain distribution and localization of IL-13Rα1, we next investigated the subcellular localization of its endogenous ligand, IL-13, in VTA neurons. In fact, we and others had previously found that, in addition to microglia, IL-13 can be produced in a fraction of neurons [15, 17]. We performed immunostaining with IL-13 antibody on the VTA brain slices and examined the staining under electron microscopy. The results revealed that IL-13 was localized pre-synaptically in vesicular structures, especially those adjacent to the active zones (Fig. 2A–D). These observations suggest the possibility that IL-13 could be released from the neuronal terminals in the VTA and thus modulate the dopaminergic neurons by acting on neuronal IL-13Rα1 in a neuropeptide-like fashion.

### Neuronal IL-13/IL-13Rα1 signaling negatively regulates the activity of midbrain dopaminergic neurons

Our observations suggest that IL-13/IL-13Rα1 signaling may play a role in regulating the activity of the ventral tegmental area dopaminergic (VTA DA) neurons. To test this hypothesis, we began by investigating the effects of recombinant IL-13 on the spontaneous firing rate of VTA DA neurons in acute slices from adult C57BL/6J mice using patch-clamp recordings. We measured the effects of 10 and 1 ng/mL of exogenously administered IL-13, within the 1–50 ng/mL range typically used to study cytokines in the brain [17, 62]. IL-13 (10 ng/mL) hyperpolarized the resting membrane potential of VTA DA neurons from −54 ± 2–−57 ± 2 mV (n = 8 cells, p < 0.05, paired t-test) and reduced the spontaneous firing rate from 3.3 ± 0.56–1.6 ± 0.4 Hz (45 ± 8% reduction, n = 18 cells, *p* < 0.0001, paired t-test, Fig. 2E–G). These effects developed slowly over 5–10 min of perfusion, partially recovered after 20 min of washout (Fig. 2F, G), and were not observed in VTA DA neurons of mice null for IL-13Rα1 **(**Fig. 2G; Supplementary Fig. 1**)**, indicating that the effects of IL-13 require IL-13Rα1. The effects of IL-13 were dose-dependent, as a lower concentration (1 ng/mL) of IL-13 failed to significantly modulate the firing rates of TH^+^ cells (96 ± 6%, n = 6 cells; p = 0.4, paired t-test). The identity of recorded neurons was confirmed as TH^+^ by post-recording immunohistochemical analysis (Fig. 2H). IL-13 did not alter resting membrane potential or firing rate of TH^-^ cells (n = 4 cells, p = 0.23, paired t-test), suggesting that the actions of IL-13 are restricted to DA neurons in VTA (Supplementary Table I). These data demonstrate that IL-13 exerts a profound inhibitory influence on dopamine neurons in the VTA, consistent with the robust expression levels of receptor in these cells.

To further confirm these findings, we examined the effects of IL-13 on calcium activity in VTA DA neurons by monitoring the fluorescence intensity changes of the calcium indicator GCaMP6s. To restrict the expression of GCaMP6s in VTA DA neurons, Cre-inducible AAV-hSyn-DIO-GCaMP6s virus was injected bilaterally into the VTA of DAT-Cre mice. Four weeks after virus injection, acute VTA-containing brain slices were prepared, and GCaMP6s fluorescence intensity was monitored. Application of IL-13 (10 ng/mL) dramatically reduced the fluorescence intensity in VTA DA neurons (Supplementary Fig. 2A, B; Supplementary Video 1). These data are consistent with the observed ability of IL-13 to decrease spontaneous activity of VTA DA neurons (Fig. 2E–H). We also tested the effects of IL-4, which, like IL-13, can also bind to the IL-13Rα1/IL-4Rα heterodimer. As expected, IL-4 (10 ng/mL) produced similar but milder effects than IL-13 (Supplementary Fig. 2C, D, G), likely due to the different differences in binding affinity [63]. Next, we tested the effects of IL-2, whose receptor subunits (IL-2Rα, IL-2Rβ, and IL-2Rγ) are expressed at much lower levels in VTA DA neurons than IL-13Rα1 and IL-4Rα **(**Fig. 1J**)**. IL-2 (10 ng/mL) had no effects on calcium activity in these neurons **(**Supplementary Fig. 2E, F, G). Taken together, these results demonstrate that IL-13 negatively regulates the activity of VTA DA neurons and that these effects required IL-13Rα1.

### IL-13 antagonizes the excitatory effects of nicotine and decreases nicotine reinforcement in rodents

The stimulatory actions of nicotine on VTA DA neurons are considered critical for the reinforcing properties of the drug [64]. Since IL-13 exerts an inhibitory action on VTA DA neurons, we hypothesized that this cytokine may oppose the stimulatory effects of nicotine. To test this, we first measured the reciprocal effects of nicotine and IL-13 on GCaMP6s fluorescence intensity in VTA DA neurons. The imaging recordings showed that IL-13 (10 ng/mL) reduced the activity of VTA DA neurons, as expected, and that the effects were partially rescued by nicotine (10 µM) (Supplementary Fig. 3A, B). Consistently, nicotine (10 μM) significantly increased the spontaneous firing rate of VTA DA neurons, and this effect was blocked by IL-13 (10 ng/mL) (Supplementary Fig. 3C, D; Supplementary Video 2). Collectively, these data demonstrate that IL-13 indeed antagonizes the stimulatory effects of nicotine on VTA DA neurons.

**Fig. 3:**
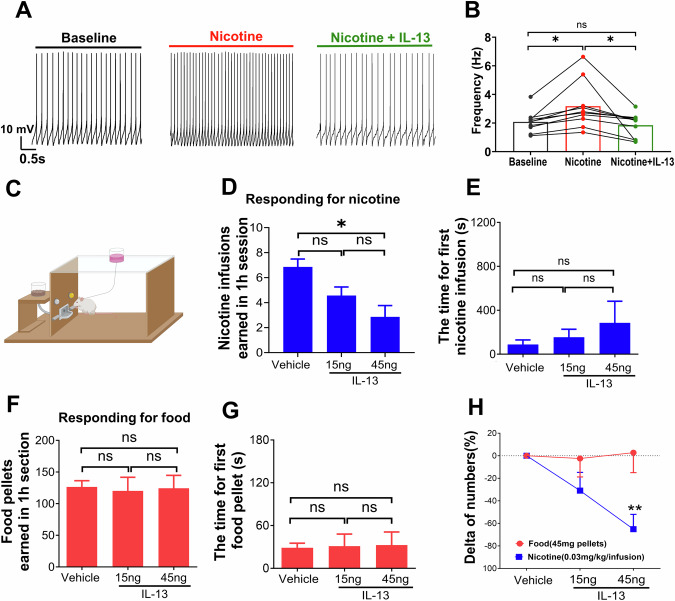
IL-13 suppresses the stimulatory effect of nicotine and local infusion of IL-13 into VTA decreases nicotine intravenous self-administration in rodents. **A** Representative traces showing the firing rate changes in VTA dopamine neurons in response to nicotine (10 μM), and then nicotine (10 μM) + IL-13 (10 ng/mL). **B** Summary of firing frequencies (n = 10 cells, from 5 mice). F(2,18) = 8.40, p < 0.01, One-way repeated measures ANOVA, for main treatment effect; *, p < 0.05; ns, not significant; post-hoc Tukey’s multiple comparisons test among means. **C** Schematic of the operant self-administration setup. **D** Infusion of exogenous recombinant IL-13 into VTA dose-dependently decreased nicotine IVSA (FR5TO20) in rats. **E** Histogram showing latency to earn the first nicotine infusion under different treatments. **F** Intra-VTA infusion of IL-13 did not alter food-reinforced responding under FR5TO20 schedule. **G** Histogram latency to earn the first food pellet under different treatments. (**D**), F (1, 7) = 8.6, p < 0.05; (**E**), F (1., 5) = 0.969, p = 0.37; (**F**), F (1., 5) = 0.058, p = 0.82; (**G**), F (1, 5) = 0.033, p = 0.8; One-way repeated measures ANOVA, for main treatment effect; *,p < 0.05; ns, not significant; post-hoc Tukey’s multiple comparisons. **H** Direct comparison of the effects of IL-13 on responding for nicotine or food rewards, expressed as percent change from baseline intake. F(2,20) = 4.64, p < 0.05, for reinforcer effect; F (1, 10) = 5.21, p < 0.05, for dose effect; F (2, 20) = 5.53, p = 0.01, for dose x reinforcer interaction effect; Two-way repeated measures ANOVA; **p < 0.01, nicotine rewards compared with food rewards after treatment with the same dose of IL-13 (45 ng); post-hoc Bonferroni test. Data are presented as mean ± SEM.

Next, we asked whether the antagonizing effects of IL-13 on nicotine would affect nicotine-related behaviors in vivo. First, we compared oral nicotine intake between IL-13Rα1 KO mice and their wild type littermates, using nicotine-containing solutions at doses commonly used to assess the motivational properties of nicotine in mice (0.16 mg/mL, free base) [65–67]. Body weights and daily water intakes were similar in both genotype (Supplementary Fig. 4A, B), but IL-13Rα1 KO mice showed significantly more licks on the nicotine solution spouts (Supplementary Fig. 4C; F (1, 10) = 12.41, p < 0.01, Two-way repeated measures ANOVA), resulting in greater nicotine consumption (Supplementary Fig. 4D). These data support our in vitro findings and suggest that IL-13Rα1 signaling regulates nicotine reinforcement in vivo. To further test this hypothesis, we examined the effects of exogenous IL-13 infusion into the VTA on intravenous nicotine self-administration (IVSA) behaviors. This was performed in male Wistar rats, which are better suited for IVSA experiments, and allowed us to test our hypothesis across species. Rats were bilaterally cannulated in the VTA and trained to self-administer intravenous nicotine infusions (0.03 mg/kg per infusion; see **Methods**) in operant chambers (Supplementary Fig. 5 and 6; Fig. 3C). Direct infusion of IL-13 (15 ng or 45 ng) into VTA decreased nicotine self-administration in a dose-dependent manner (F (1, 7) = 8.6, p < 0.05, One-way repeated measures ANOVA; Fig. 3D). *Post-hoc* tests showed a significant reduction in nicotine intake at 45 ng IL-13 (p < 0.05), but not 15 ng. IL-13 infusions (either at 15 ng or 45 ng) did not significantly alter the latency to earn the first nicotine infusion (F(1, 5) = 0.969, p = 0.37, One-way repeated measures ANOVA; Fig. 3E), suggesting that the treatment affected nicotine’s rewarding effects rather than motor or task-related performance [68]. The suppressive effect of IL-13 on nicotine intake was blocked by co-infusion of a cocktail of Ruxolitinib and Rapamycin (Supplementary Fig. 7), which inhibit the JAK/STAT and the PI3K/AKT/mTOR pathways, respectively –both previously shown to block the neuronal action of IL-13 [42]. Moreover, intra-VTA infusions of IL-13 (15 or 45 ng) did not affect operant responding for food or the latency to earn the first food pellet (Fig. 3F, G) [69]. A direct comparison of IL-13’s on responding for nicotine versus food rewards, expressed as percent change from baseline, revealed that 45 ng IL-13 significantly decreased responding for nicotine but not food (p < 0.01, Bonferroni post-test after a significant interaction effect in Two-way repeated measures ANOVA; Fig. 3H). Taken together, these results strongly suggest that neuronal action of IL-13 signaling reduces nicotine reinforcement.

### IL-13 decreases I_h_ current amplitudes and impaired the voltage dependence of I_h_ activation in dopamine neurons

**Fig. 4:**
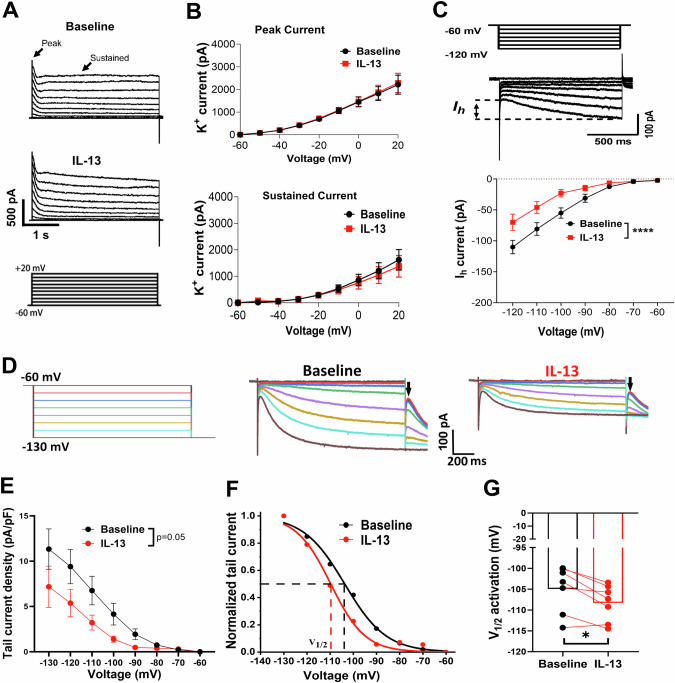
IL-13 inhibits HCN channels in the VTA dopamine neurons. **A,** **B** Representative traces (**A**) and summary data **(B)** of voltage-gated potassium channel–mediated currents in VTA DA neurons before and after treatment with IL-13 (10 ng/mL) (n = 7 cells, from 4 mice). **C** Representative trace (top) and summary data (bottom) of *I*_*h*_ currents before and after treatment with IL-13 (n = 11 cells, from 6 mice). ****, p < 0.0001; Two-way repeated measures ANOVA, main treatment effect. **D** Representative traces of tail currents of HCN channels before and after treatment with IL-13. The tail currents were evoked by a series of voltage steps as indicated on the left. Arrows indicate the tail currents. **E** Tail current density plotted versus the preceding test potential. Tail current density was calculated by tail current amplitudes divided by the membrane capacitance of each neuron. F(1, 6) = 5.757, p = 0.05, Two-way repeated measure ANOVA, for main treatment effect. **F** *I*_*h*_ activation curves fitted with Boltzmann functions. **G** IL-13 induced a significant hyperpolarizing shift of the half activation potential (V_1/2_) of HCN channels. *, p = 0.013; paired t test; n = 7 cells, from 4 mice. Data are presented as mean ± SEM.

Next, we sought to elucidate the potential ionic mechanisms underlying the inhibitory effects of IL-13 on VTA DA neurons. Emerging evidence suggests that voltage-gated potassium channels and HCN (Hyperpolarization-activated, cyclic nucleotide-gated) channels are two critical regulators of DA neuronal activity [70–72]. Thus, we hypothesized that these channels may be involved in the effects observed with IL-13 treatment. To test this, we performed whole-cell patch-clamp recordings on the VTA TH^+^ neurons from acute brain slices of C57BL/6J mice. Both potassium currents, mediated by voltage-gated potassium channels, and the cation current (*I*_*h*_), mediated by HCN channels, were assessed. We found that the same concentration of IL-13 (10 ng/mL), which inhibited the firing rate of dopaminergic neurons, did not significantly affect the peak or sustained potassium currents (Fig. 4A, B), but decreased the amplitude of *I*_*h*_ currents (F(1, 10) = 51.23, p < 0.0001; Two-way repeated measures ANOVA) **(**Fig. 4C**)**. These data suggest that IL-13 modulates the activity of dopamine neurons via its effects on HCN channels. To test this hypothesis in vivo, we used optogenetics to stimulate IL-13^+^ neurons. IL-13-Cre::Ai32 mice, expressing the light-sensitive opsin channelrhodopsin-2(ChR2) in the IL-13 expressing cells, were generated by crossing IL-13-Cre with Ai32 mice. Consistent with presynaptic localization of IL-13 (Fig. 2), ChR2-positive terminals were found in the VTA area, but no positive cell bodies were observed (Supplementary Fig. 8A). Whole-cell patch-clamp recording was performed in the presence of kynurenic acid (250 μM) and bicuculine (10 μM) to block excitatory and inhibitory synaptic transmissions, thus isolating the effects of endogenous neuronal IL-13. Stimulation with 20 s constant blue light (470 nm) reduced the firing rate of the VTA DA neurons (Supplementary Fig. 8B, C**;** p < 0.01, paired t test), as well as the amplitudes of the *I*_*h*_ current (Supplementary Fig. 8D**;** p < 0.05, Two-way repeated measures ANOVA). These findings indicate that activation of endogenous IL-13 negatively regulates the activity of VTA DA neurons and the HCN channels. Thus, HCN channels represent a key target of IL-13 signaling in modulating the activity of VTA DA neurons.

To gain mechanistic insight into the action of IL-13, we further investigated how it affects the voltage dependence of *I*_*h*_ activation. To this end, tail current amplitudes at −130 mV were measured following a series of hyperpolarizing voltage steps to construct *I*_*h*_ activation curves (Fig. 4D). First, we observed that IL-13 application significantly decreased the tail current density (tail current amplitude/cell capacitance) in the VTA DA neurons compared with pre-treatment (Fig. 4E) (F (1, 6) = 5.757, p = 0.05; Two-way repeated measures ANOVA, for treatment effect). The tail current amplitudes were then plotted as a function of proceeding test potentials and were fitted with a Boltzmann function to generate *I*_*h*_ activation curves **(**Fig. 4F**)**. We found that IL-13 significantly shifted the half-activation potential (V_1/2_) to more hyperpolarized potentials (p = 0.013; paired t test). Taken together, these results demonstrate that IL-13Rα1 signaling inhibits the activation of HCN channels in VTA DA neurons, providing a cellular mechanism for the observed inhibitory effects of IL-13.

### The action of IL-13 on VTA DA neurons requires PI3K/AKT signaling pathway and HCN channels

**Fig. 5:**
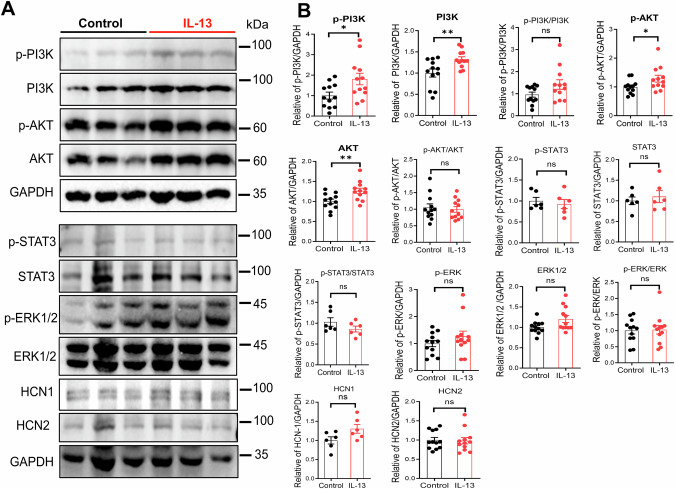
IL-13 activates the PI3K/AKT signaling pathway in the VTA. **A** Western blots showing PI3K, p-PI3K, AKT, p-AKT, STAT3, p-STAT3, ERK1/2, p-ERK1/2, HCN1, and HCN2 expression in VTA extracts from brain slices perfused for 20 min with aCSF alone (Control) or with aCSF containing IL-13 (10 ng/mL). **B** Quantification of protein expression. n = 6 or 12 mice per group; *p < 0.05, **p < 0.01; ns, not significant; unpaired t test. Data are presented as mean ± SEM.

**Fig. 6:**
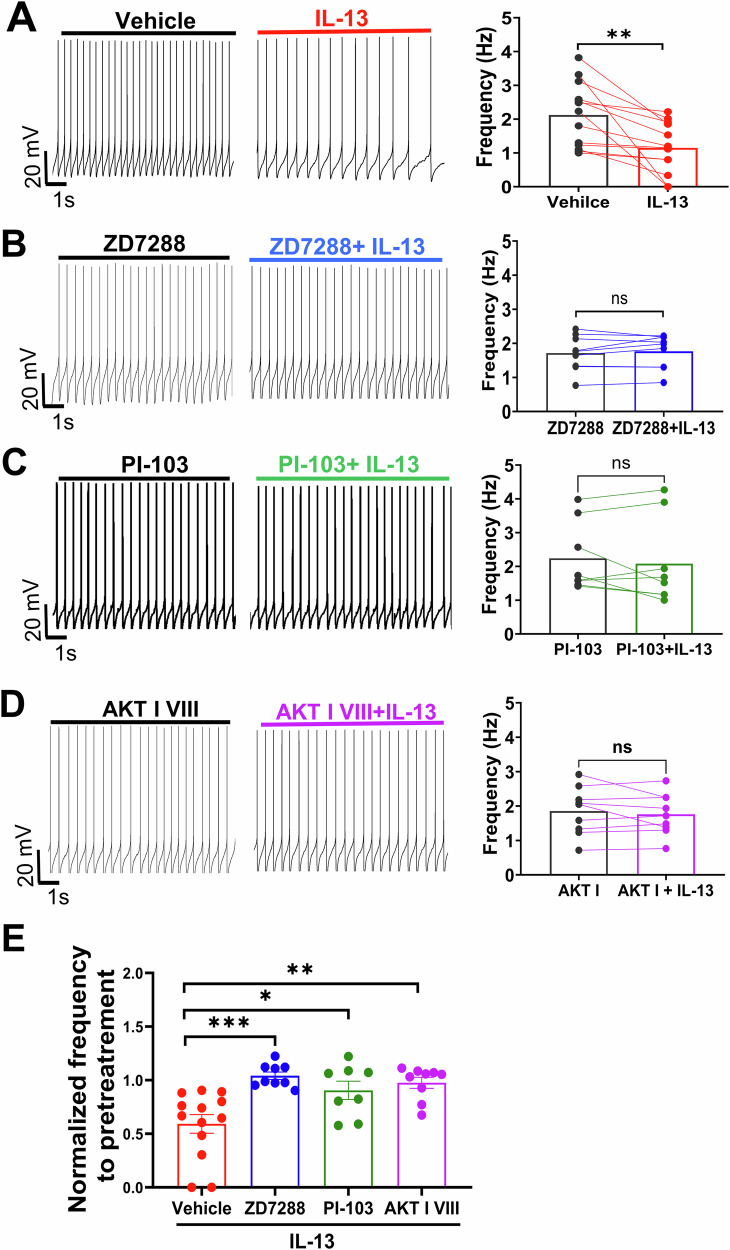
The inhibitory effect of IL-13 on VTA dopamine neuron activity requires PI3K/AKT signaling and HCN channel function. **A**–**D** Representative traces and summary data showing the effects on VTA DA firing rate of IL-13 (10 ng/mL) alone (**A**), HCN channel blocker ZD7288 (10 μM) + IL-13 (**B**), PI3K inhibitor PI-103 (1 μM) + IL-13 (**C**), and AKT inhibitor III (1 μM) + IL-13 (**D**). The pretreatment time was 20–30 min right before each recording. (**A**), **, p < 0.01, n = 13 cells, from 6 mice; (**B**), ns, n = 9 cells, from 4 mice; (**C**), ns, n = 8 cells, from 4 mice; (**D**), ns, n = 9 cells, from 4 mice; ns, not significant, paired t test. **E** Direct comparison of the suppression effects of IL-13 with and without inhibitors. p < 0.0001, one-way ANOVA, for main treatment effect; *p < 0.05, **p < 0.01, ***p < 0.001, post-hoc Tukey’s multiple comparisons test among means. Data are presented as mean ± SEM.

Next, we investigated the signaling pathway mediating the inhibitory effects of IL-13 on the *I*_*h*_ current. To this end, we performed Western blotting on VTA tissue extracts obtained from acute brain slices perfused with either artificial cerebrospinal fluid (aCSF, control) or with aCSF containing IL-13 (10 ng/mL). IL-13 treatment significantly elevated the levels of p-PI3K, PI3K, p-AKT and AKT, without affecting the JAK/STAT or the ERK1/2 pathways (Fig. 5A, B). IL-13 did not change the levels of HCN1 or HCN2 (Fig. 5A, B**)**, the two major HCN subtypes in the VTA [71]. These data suggest that the inhibitory effect of IL-13/IL-13Rα1 on the activation of HCN channels is mediated by enhancing the PI3K/AKT signaling, rather than by changes in HCN protein expression. This is consistent with previous reports demonstrated that HCN function is regulated by PI3K/AKT signaling rather than HCN protein abundance [73]. To further examine the roles of PI3K/AKT, the effects of IL-13 were assessed pharmacologically in whole-cell patch-clamp recordings on VTA DA neurons in acute brain slices from adult C57BL/6J mice. As expected, IL-13 significantly decreased the activity of these neurons (Fig. 6A; p < 0.01, paired t test). However, this inhibitory effect was blocked by pretreatment with the HCN channels blocker ZD7288 (10 μM) (Fig. 6B, F), the PI3K inhibitor PI-103 (1 μM) (Fig. 6C, F), or the AKT inhibitor VIII (1 μM) (Fig. 6D, F). Thus, these findings provide strong evidence that IL-13/IL-13Rα1 signaling inhibits VTA DA neuronal activity through a PI3K/AKT-dependent modulation of HCN channels.

## Discussion

We provide data indicating that the cytokine IL-13 acts in a neuromodulator-like fashion to lower the firing rate of midbrain dopaminergic neurons, thereby decreasing the excitatory effects of nicotine and its associated rewarding behaviors. Furthermore, we provide evidence that these actions require IL-13Rα1, activation of the PI3K/AKT pathway, and the hyperpolarization-activated cyclic nucleotide-gated (HCN) channel.

Understanding the mechanisms by which cytokines regulate neuronal survival and function is crucial for elucidating their roles in the central nervous system. Several laboratories have demonstrated that these molecules can act indirectly by regulating prostaglandin production or modulating microglial activity [9, 74–76]. However, evidence that cytokine receptors are expressed in neurons suggests that direct actions are also possible [12, 13, 77]. For instance, we previously reported that IL-13Rα1 was primarily expressed in TH^+^ neurons of the substantia nigra pars compacta and ventral tegmental area (VTA) in the healthy brain, and was induced by stress in the locus coeruleus [15, 16]. This distribution suggests that IL-13Rα1 may specifically influence catecholaminergic neurons and their functions.

Here, we confirm that IL-13Rα1 and IL-4Rα are expressed in midbrain dopaminergic neurons, with these receptors being the most abundantly expressed immune receptors in these cells. In contrast, two potential competitors of IL-13 signaling—the IL-13Rα2, which lacks a cytoplasmic domain, and the IL-2rg (also known as γc or CD132), which can sequester IL-4Rα, were barely detectable. We have previously shown that, during restraint stress or peripheral challenge with bacterial lipopolysaccharides, IL-13 is inducible in microglia as well as in neurons [14–16]. Here, we confirm neuronal expression of IL-13 and provide evidence of its localization in vesicular structures near the presynaptic active zone. Although the mechanisms underlying IL-13 secretion or release remain to be determined, our data using IL-13-Cre::Ai32 optogenetic models indicate that neuronal stimulation mobilizes endogenous IL-13, affecting the activity of VTA DA neurons. These observations are consistent with recent findings demonstrating presynaptic localization of IL-13 in both glutamatergic and GABAergic cortical neurons [17]. Electrophysiological recordings of TH^+^ neurons in the VTA of mouse brain slices revealed that IL-13 significantly reduced their firing rate, and this effect required IL-13Rα1. The IL-13Rα1/IL-4Rα complex is a type I cytokine receptor, which lacks ion channel function but can activate at least two signal transduction pathways: JAK/STAT and PI3K/AKT/mTOR. Thus, IL-13Rα1 acts indirectly to regulate firing rate of dopaminergic neurons. We found that IL-13 stimulated the PI3K and AKT pathway in VTA slices, which are known to regulate HCN channel, a critical modulator of the firing of VTA DA neurons. While the exact mechanisms by which PI3K/AKT activation affects HCN channel activity remain to be determined, our data show that IL-13 elevated both total and phosphorylated PI3K and AKT within 20 min. One possibility is that IL-13 promotes the translation of PI3K and AKT. We also demonstrated that pharmacological inhibition of PI3K, AKT, or HCN channels blocked the effects of IL-13. Taken together, these data suggest that IL-13 modulates the activity of dopaminergic neurons via direct activation of neuronal IL-13Rα1 and PI3K/AKT signaling, which in turn affects the function of HCN channels. As observed in models of neurodegeneration, the neuromodulatory effects of IL-13 may differ depending on the neuronal types. In fact, in cortical neurons expressing IL-13Rα1, IL-13 regulates plasticity by enhancing the phosphorylation of NMDA and AMPA receptors, thereby increasing synaptic activity and neuronal firing [17]. Despite these differences, all data support the conclusion that IL-13 functions as a neuromodulator, with its presynaptic localization suggesting the possibility of a trans-synaptic mode of action.

Clinical and experimental evidence indicates that cytokines produced during infection or tissue damage can affect behavior [10]. The concept of *sickness behavior* was first postulated by Neil Miller in 1964 as a motivational state mediated by endogenous factors produced during disease [78]. Thus, it is not surprising that several findings indicate that regulating immune functions can affect addiction, itself a motivational state [5, 6]. Here we performed experiments to test the hypothesis that IL-13 and IL-13Rα1 signaling regulate the rewarding effects of nicotine. Nicotine exerts stimulatory effects on VTA DA neurons, which are thought to be critical for the reinforcing properties of the drug [64]. Consistent with the inhibitory effects of IL-13Rα1 signaling on VTA DA neurons, IL-13 opposed the stimulatory effects of nicotine in brain slices, suggesting IL-13 signaling may negatively regulate nicotine’s rewarding effects. To test whether IL-13 acts in the VTA to regulate nicotine intake, we infused IL-13 directly into the VTA and measured its effect on nicotine self-administration. We found that exogenous IL-13 reduced nicotine self-administration behavior in a dose-dependent manner. These effects were blocked by the pharmacological inhibition of IL-13Rα1 signaling and were not secondary to disruptions in behavioral performance.

Previous studies have demonstrated that central IL-13 levels are elevated during restraint stress as well as following peripheral administration of bacterial lipopolysaccharides, a surrogate of infection [14–16]. This suggests that IL-13 production during immune activation may reduce mesocorticolimbic dopaminergic function via IL-13Rα1. The resultant reduction in reward system function is consistent with the change of motivational behavior associated with sickness behavior and could explain the reduced drug consumption observed in animals that develop infections during intravenous self-administration experiments.

One limitation of this study is that experiments were conducted only in male animals, and it is possible that the findings may differ in females. For instance, IL-13 and estrogen have been reported to exert opposing effects on MHCII regulation in macrophages [79], and their combined effects on dopaminergic neurons remain to be investigated. In addition, since *Il3ra1* is located on the X chromosome, genetic variants of this gene may contribute to sex-specific phenotypic differences. Another limitation is that we did not investigate whether nicotine use affects IL-13 or its receptor. Recent work in rodents showed that nicotine did not alter IL-13 or IL-13R in the VTA [80], nor the level of plasma IL-13 [81], but the effects of nicotine withdrawal remain to be evaluated. Future studies are needed to fully elucidate the role of IL-13/IL-13Rα1 signaling in the development of nicotine addiction and its possible translational relevance. An intriguing possibility is that a human genetic variant in the *IL13* promoter, previously linked to an increased risk of smoking-related lung disease, may affect nicotine addiction and consumption [49]. It will also be important to determine the specific conditions under which IL-13 exerts its hypodopaminergic functions, identify the neurons providing synaptic regulation of IL-13Rα1 in the VTA, and assess the relative contributions of neuronal versus microglia IL-13. We previously showed that IL-13 levels in microglia and neurons are elevated during neuroinflammation or stress, making it plausible that IL-13 contributes to the hypodopaminergic phenotype in such conditions. However, IL-13 is not the only cytokine produced during inflammation or stress that may also affect dopaminergic functions, and this remains an area for further investigation. Among the cytokines to investigate is IL-4, which can activate IL-13Rα1/IL-4Rα. Here, we focused primarily on IL-13 in this study because, under the experimental conditions used, we did not detect significant levels of IL-4 [14, 16], though we found that exogenous IL-4 did affect the activity of dopaminergic neurons, albeit with a lower intensity than IL-13. This difference may be due to the fact that, unlike IL-13, the main source of IL-4 in the brain is represented by infiltrating T cells [14, 15, 17, 23–25], and IL-4 binds with higher affinity to the IL-4Rα/IL-2rg complex, which was not investigated here. However, mice lacking IL-4 or IL-4Rα have impaired learning and memory, indicating that this cytokine may also regulate neuronal functions [82]. Finally, although we observed predominant IL-13Rα1 expression in VTA TH^+^ neurons and found no effects of IL-13 in the TH^-^ neurons, we cannot rule out the possibility that IL-13Rα1/IL-4Rα is expressed in GABAergic neurons within the VTA.

In summary, our findings reveal neuronal IL-13/IL-13R signaling as a key neuromodulator of dopaminergic function in the VTA and provide a model of how cytokines may act directly on neurons to regulate neuronal function and behavior. Our results also suggest that the mode of IL-13 (or IL-4) production and release in the CNS may affect motivation and vulnerability to nicotine addiction.

## Supplementary information

Supplemental Material

Suppl Video 1

Suppl Video 2

## Data Availability

Analyzed data and images reported in this paper will be shared by the corresponding authors upon request.
