# Supplementary material for: Interleukin 13 signaling modulates dopaminergic functions and nicotine reward in rodents
Source: Mol Psychiatry. 2025 Aug 7;31(2):622–34. doi: 10.1038/s41380-025-03137-3 (PMC12542912; doi:10.1038/s41380-025-03137-3)
Supplement: Supplementary file 1 — Supplemental Material [file 41380_2025_3137_MOESM1_ESM.pdf]

Supplementary information

Supplementary Materials and Methods

Animals

DAT-Cre mice (Jax #006660), IL-13-Cre mice (Jax #017353) and Ai32 mice (Jax #012569) were used. The IL-13-Cre::Ai32 mice used in optogenetic experiments were generated by pairing IL-13-Cre mice with Ai32 mice. IL-13Rα1 knock-out (IL-13Rα1 KO) mice used for breeding were purchased from Shanghai Model Organizations (Cat# NM-KO-190457l; Shanghai, China). IL-13Rα1 KO mice were then crossed with C57BL/6J mice, the offspring male IL-13Rα1 KO mice and their WT littermates were used for experiments. All mice were housed in groups of 5 per cage at 22-25°C and 30-40% humidity on a 12-h light/dark cycle, with food and water available *ad libitum*.

Ca^{2+} imaging on acute brain slices

The genetically encoded Ca^{2+} indicator GCaMP6s was used for Ca^{2+} imaging of VTA in the brain slice. 200-250 nL of AAV-hSyn-DIO-GCaMP6s virus (2-10 × 10^{12} viral particles/mL) (BrainVTA Co., Ltd., China) was injected bilaterally into VTA of DAT-Cre mice. Four weeks after the injection, acute brain slices containing VTA were prepared as for patch-clamp recordings. The in vivo imaging experiments were performed using a Bruker Investigator two-photon system equipped with a DeepSee Ti: sapphire laser (Spectra Physics) tuned to 920 nm. For Ca^{2+} imaging, we chose Resonant Galvo model for long-duration imaging. The images were collected at a depth of 50–200 µm at frame rates of 2 Hz, and a resolution of 1024 × 1024 pixels using a 16X objective (NA = 0.8) immersed in artificial cerebrospinal fluid and with a 1 × digital zoom. There was no marked difference in image quality at different depths of brain slice. Image acquisition was performed using Bruker Prairie View software. The imaging parameters were chosen to allow repeated imaging of the same cells without causing damage. The same acute brain slice was first recorded for 5 min for baseline intensity, then subjected to different treatments and recorded for 5 min for each treatment. The treatments were IL-13 (10 ng/mL), IL-4 (10 ng/mL), IL-2 (10 ng/mL), nicotine (10 µM). The concentration of the IL-13 was chosen based on previous dose response studies [1-4]. We recorded 1-2 VTA containing brain slices per mouse from at least 3 mice for an

average of 120 cells was recorded for each treatment. The experimental conditions were kept the same throughout all the imaging sessions to ensure proper calculation of image contrast. The intensity of the cells was analyzed with ImageJ software. The averaged intensity of each cell during the 5 min of baseline and treatments were normalized to the averaged intensity at the baseline.

Oral nicotine-containing solution intake measurements

The experiment was conducted with an adapted Vogel test system from Panlab, Harvard apparatus. The setup consists of 8 standard test cages, and each equipped with a bottle and a lickometer nipple for real-time measurement of solution licking behavior. On Day 0 , mice that had been deprived of water for 24 hours were individually placed in separate cages. They were allowed free access to water for 4h as the habituation process. From Day 1 on to Day 10, water was placed with nicotine solution (160 μg/mL nicotine + 0.2% saccharin) in the bottles of the test cages. The number of licks at the spout, and the amount of nicotine solution intake of each mouse were recorded in the 4h sessions. The concentrations of nicotine and saccharin were chosen based on previous studies. Saccharin was added to mask the bitter taste of nicotine. Mice were allowed full free access to water and food in their home cages in the nicotine intake measurement days.

Optogenetic stimulation with electrophysiology recordings

Acute brain slices from mice (IL-13-cre::Ai32, male, 8-10 weeks) were prepared as previously described[5]. The VTA slices were recovered in artificial cerebrospinal fluid (ACSF) at 32℃ for 30 min followed by incubation in ACSF at room temperature for an additional 30 min before recordings. The brain slices were then transferred to a recording chamber on a microscope stage (BX51WIF, Olympus, Japan) and were continuously perfused with oxygenated ACSF maintained at 32°C at a rate of 2–3 mL/min. VTA area was visually located using a 5x objective, within which neurons were identified using a 40x water immersion objective based on observed fluorescence through a green excitation light source (587 nm, pE-300, CoolLED, UK). Whole-cell patch-clamp recordings from VTA neurons were performed as described before. Briefly, recording pipettes were pulled from borosilicate glass (BF150-86-10, Sutter Instruments, USA) on a Flaming/Brown

micropipette puller (Model P-1000, Sutter Instruments, USA), and had resistance of 4-8 MΩ when filled with (in mM): 130 K-gluconate, 10 KCl, 10 HEPES, 1 EGTA, 2 MgATP, 0.4 NaGTP and 2 MgCl2 (300 mOsm, pH adjusted to 7.2). Hyperpolarization-activated cation currents (*Ih*) were evoked by applying voltage steps from −60 mV to −130 mV in 10 mV increments, from a holding potential of −60 mV. Spontaneous action potentials (APs) firing at resting membrane potential were recorded in the presence of Bicuculline (10 µM) and Kynurenic acid (250 µM) to block excitatory and inhibitory synaptic transmission. To access responses of VTA neurons induced by optogenetic stimuli, optically-evoked APs and poststimulation spontaneous APs were recorded by delivering continuous 470 nm optical stimulation (20 s duration). Electrophysiological signals were amplified using a Multiclamp 700B amplifier (Axon Instruments, Molecular Devices, CA). Data were low-pass filtered at 10 kHz and digitized at 20 kHz using Clampex 11.1 software (Molecular Devices, CA) in conjunction with a Digidata 1550B data acquisition system (Axon Instruments, Molecular Devices, CA).

Supplementary Table

TH- neurons (n=4)	RMP	Firing rate
**Control**	-59.8 ± 3.2 mV	2.0 ± 0.5 Hz
**IL-13 (10 ng/mL)**	-58.1 ± 2.5 mV	2.2 ± 0.7 Hz

Supplementary Table I. Electrophysiological properties of TH^{-} neurons treated with vehicle (Control) or IL-13.

Supplementary Figures and Legends

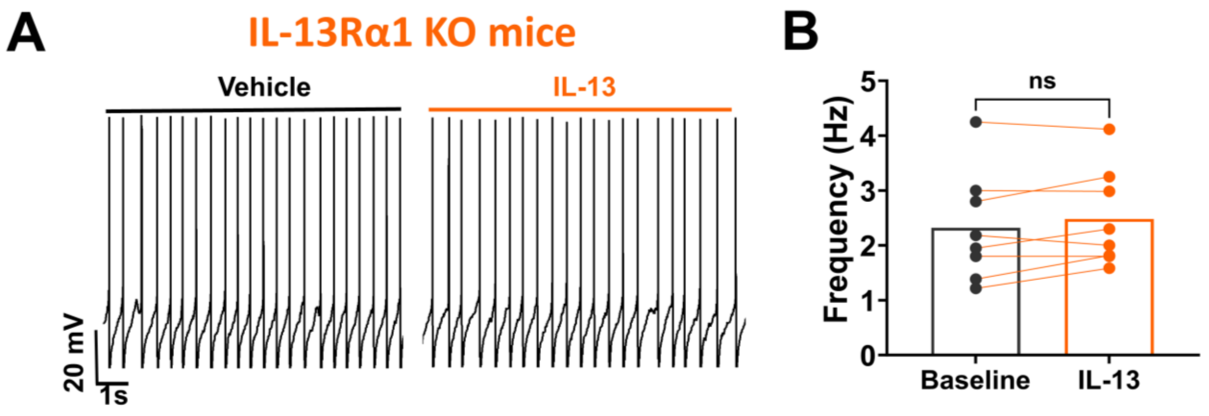

Supplementary Figure 1. IL-13 application does not change the firing rate of VTA DA neurons in the IL-13Rα1 knock-out mice.

(**A-B**) Representative traces (A) and summary data (B) show the effects of IL-13 on the firing rate of dopamine neuron from adult IL-13Rα1 knockout mice. n=8 cells, from 4 mice. ns, not significant, paired t test. Data are presented as mean ± SEM.

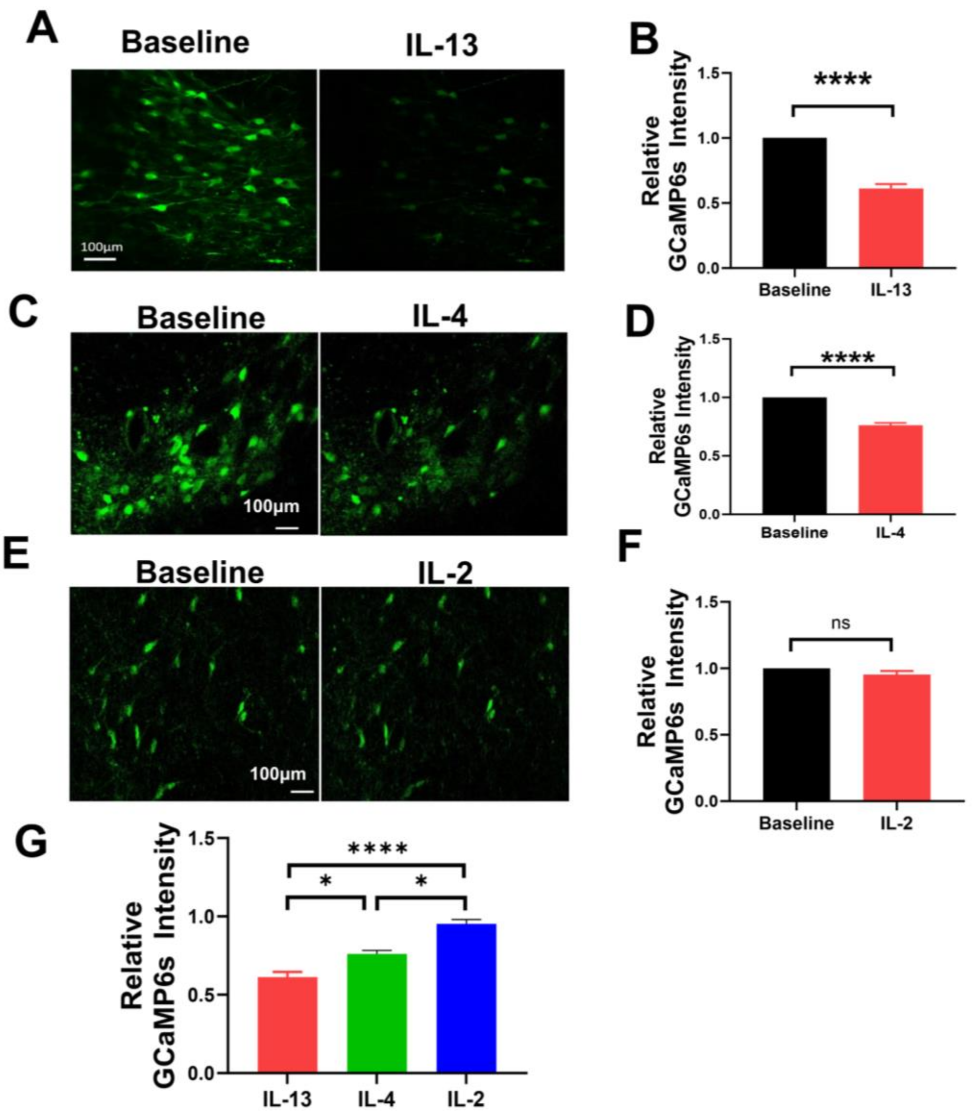

Supplementary Figure 2. IL-13 and IL-4, but not IL-2, reduce GCaMP6s fluorescence intensity in VTA dopamine neurons.

(A-B) Representative fluorescence images for GCaMP6s intensity in the VTA response to application of IL-13 (10 ng/mL) (A), and summary of the intensity of GCaMP6s responses that was normalized to baseline (B). n=6 slices from 3 mice, 197 neurons were analyzed; ****, $p<0.0001$, paired t test. **(C-D)** Representative fluorescence images for GCaMP6s intensity in the VTA response to application of IL-4 (10 ng/mL) (C), and summary of the intensity of GCaMP6s responses that was normalized to baseline (D). n=3 slices from 3 mice, 69 neurons were analyzed; ****, $p<0.0001$, paired t test. **(E-F)** Representative fluorescence images for GCaMP6s intensity in the VTA response to application of IL-2 (10 ng/mL) (E), and summary of the intensity of GCaMP6s

responses that was normalized to baseline (F). n=3 slices from 3 mice with 49 neurons were analyzed. ns, not significant; paired t test. **(G)** Group summary of the intensity of GCaMP6s responses that was normalized to baseline. F (2, 306) = 15.63, $p<0.001$; One-way repeated measures ANOVA, for main treatment effect; *, $p<0.05$, ****, $p<0.0001$, post-hoc Tukey's multiple comparisons test among means. Data are presented as mean± SEM.

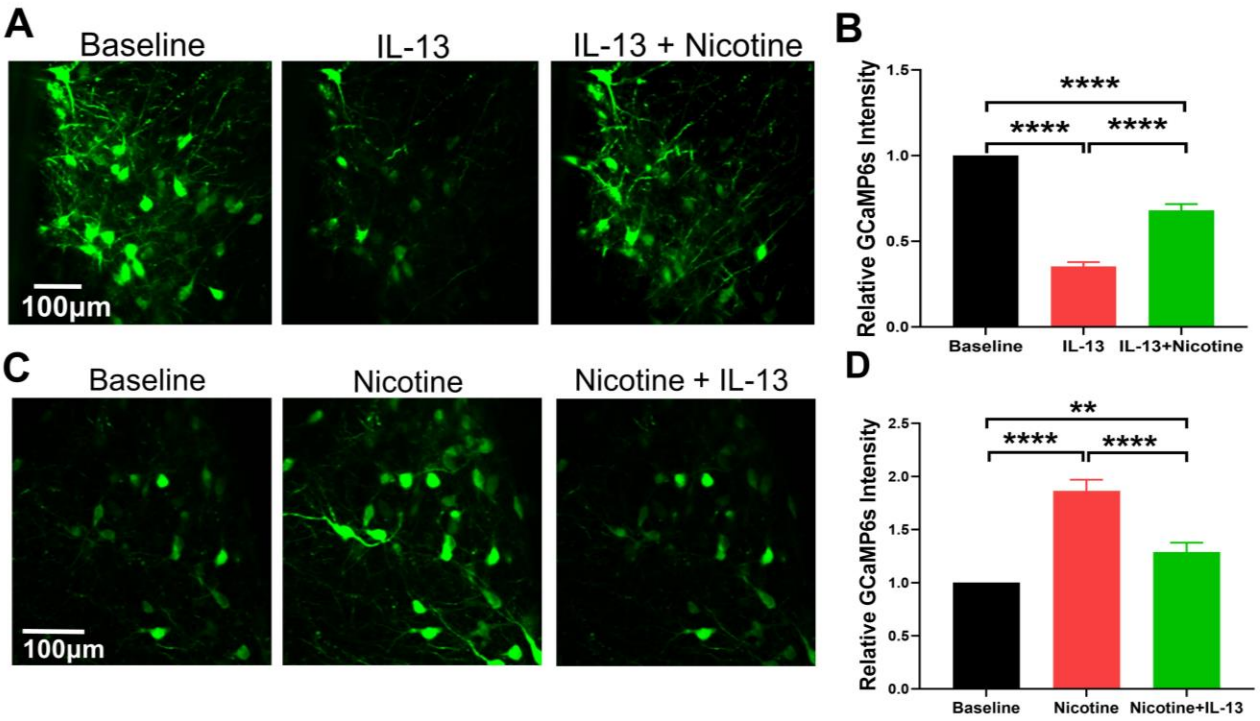

Supplementary Figure 3. IL-13 counteracts the stimulatory effect of nicotine on VTA dopamine neurons.

(A-B) Representative fluorescence images for GCaMP6s intensity in the VTA response to application of IL-13 (10ng/mL) alone, or together with nicotine (10 µM) (A), and summary of the intensity of GCaMP6s responses that was normalized to baseline (B). Note that the intensity of GCaMP6s decreased after IL-13 application and was partially rescued by nicotine. n=6 slices from 3 mice, 163 neurons were analyzed; F (1.721, 148.0) = 210.2, p<0.0001, One-way repeated measures ANOVA, for main treatment effect; ****, p<0.0001, post-hoc Tukey's multiple comparisons test among means. (**C-D**) Representative fluorescence images for GCaMP6s intensity in the VTA response to application of nicotine (10 µM) alone, or together with IL-13(C), and summary of the intensity of GCaMP6s responses that was normalized to baseline (D). n=3 slices from 3 mice, 89 neurons were analyzed; F (1.868, 302.6) = 37.35, One-way repeated measures ANOVA, for main treatment effect; **, p<0.01, ****, p<0.0001, post-hoc Tukey's multiple comparisons test among means. Data are presented as mean± SEM.

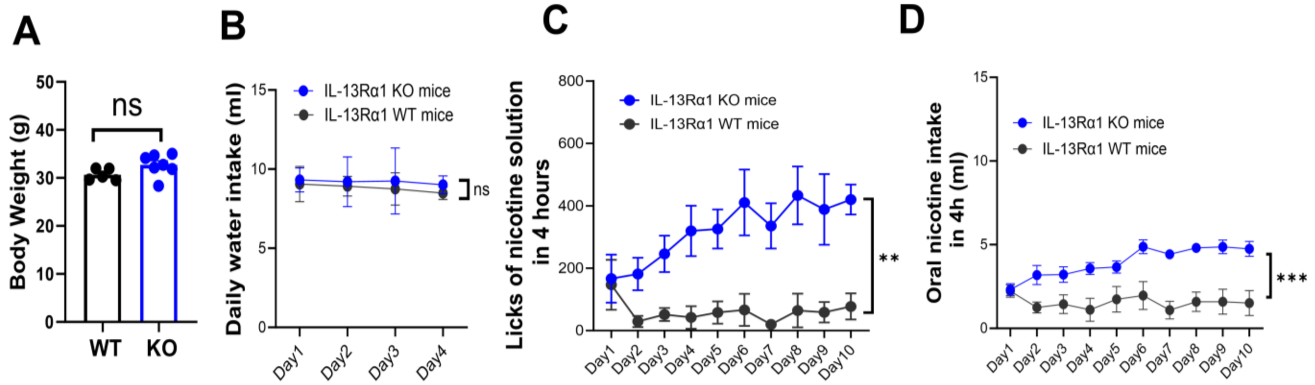

Supplementary Figure 4. IL-13Rα1 knockout mice exhibit increased nicotine consumption compared to wild-type littermates.

(A) The body weight of IL-13Rα1 knockout mice (n=7 mice) and WT mice (n=5 mice). ns, not significant, unpaired t test. **(B)** Daily water consumption volume in the IL-13Rα1 knockout mice (n=7 mice) and their WT littermates (n=5 mice) over days. ns, not significant; Two-way repeated measures ANOVA, for genotype effect. (**C-D**) IL-13Rα1 knockout mice consumed much more nicotine solution (0.16mg/mL, free base) compared with their WT littermates in a daily 4h tests over days. C, licks number for nicotine-containing solution. $F (1, 10) = 12.41$, **, $p<0.01$, Two-way repeated measures ANOVA, for genotype effect. D, nicotine-containing solution intake volume. $F (1, 10) = 31.68$, ***, $p<0.01$; Two-way repeated measures ANOVA, for genotype effect. Data are presented as mean± SEM.

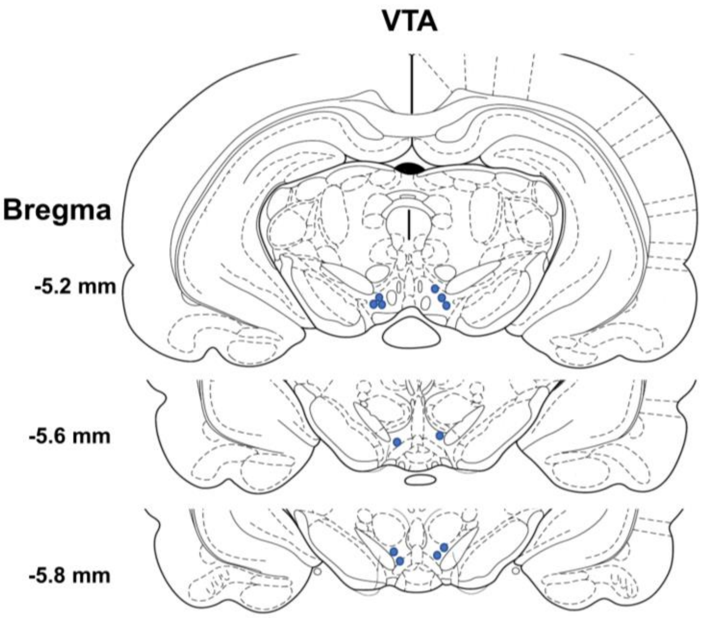

Supplementary Figure 5. Schematic representation of the injection tips in the VTA in coronal sections of rats. The locations of the injection tips are represented by blue circles.

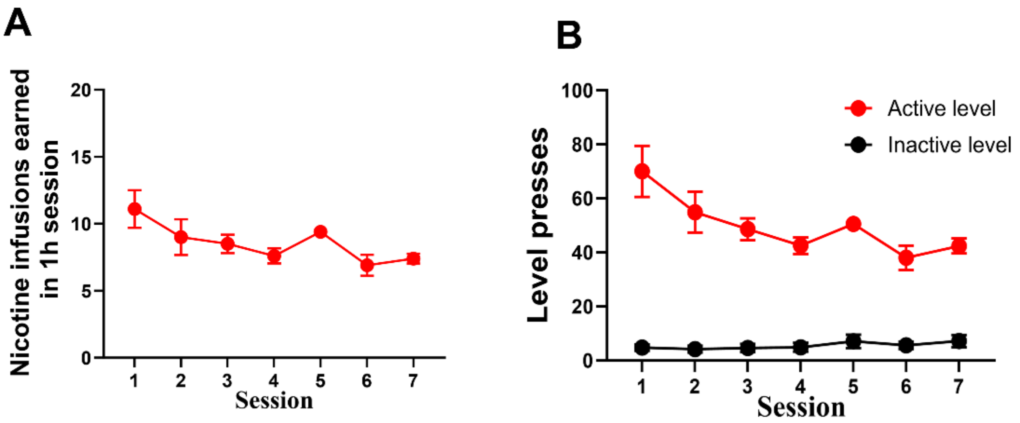

Supplementary Figure 6. Nicotine self-administration behavior during the training sections. **(A)** Number of nicotine infusions during 1-hour daily sessions over 7 consecutive days. **(B)** Number of active and inactive lever presses during the same training sessions. Data are presented as mean± SEM.

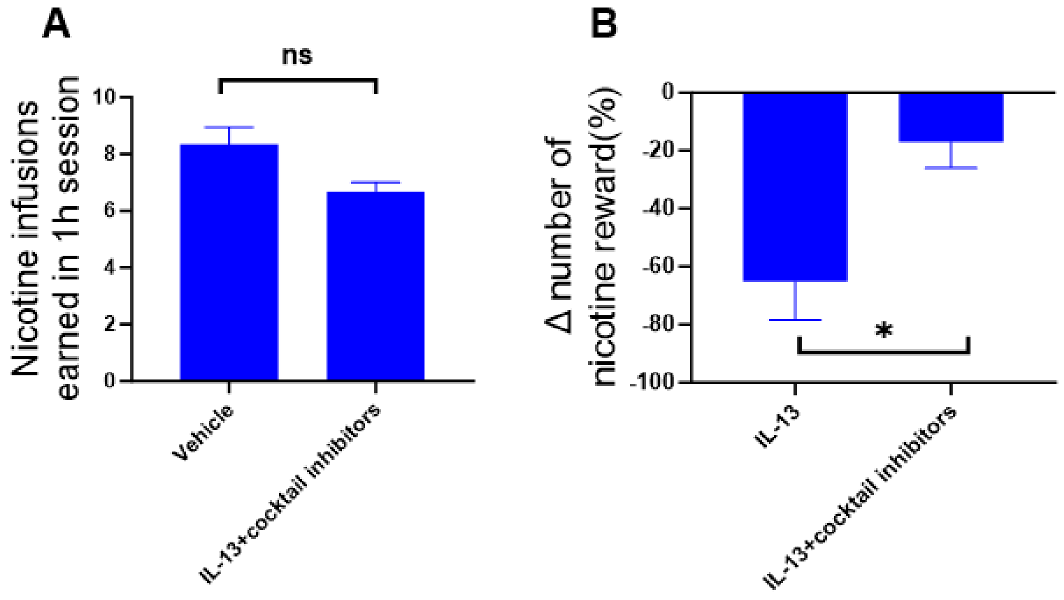

Supplementary Figure 7. Pharmacological inhibition blocks IL-13-mediated suppression of nicotine reward.

(A) Co-Infusion of IL-13 with a cocktail of inhibitors (Rapamycin and Ruxolitinib) into VTA did not significantly alter nicotine IVSA behavior in rats. (**B**) Direct comparation of the effects of IL-13 vs IL-13+cocktail inhibitors on nicotine IVSA behavior. ns, not significant; *p<0.05, paired t test. Data are presented as mean± SEM.

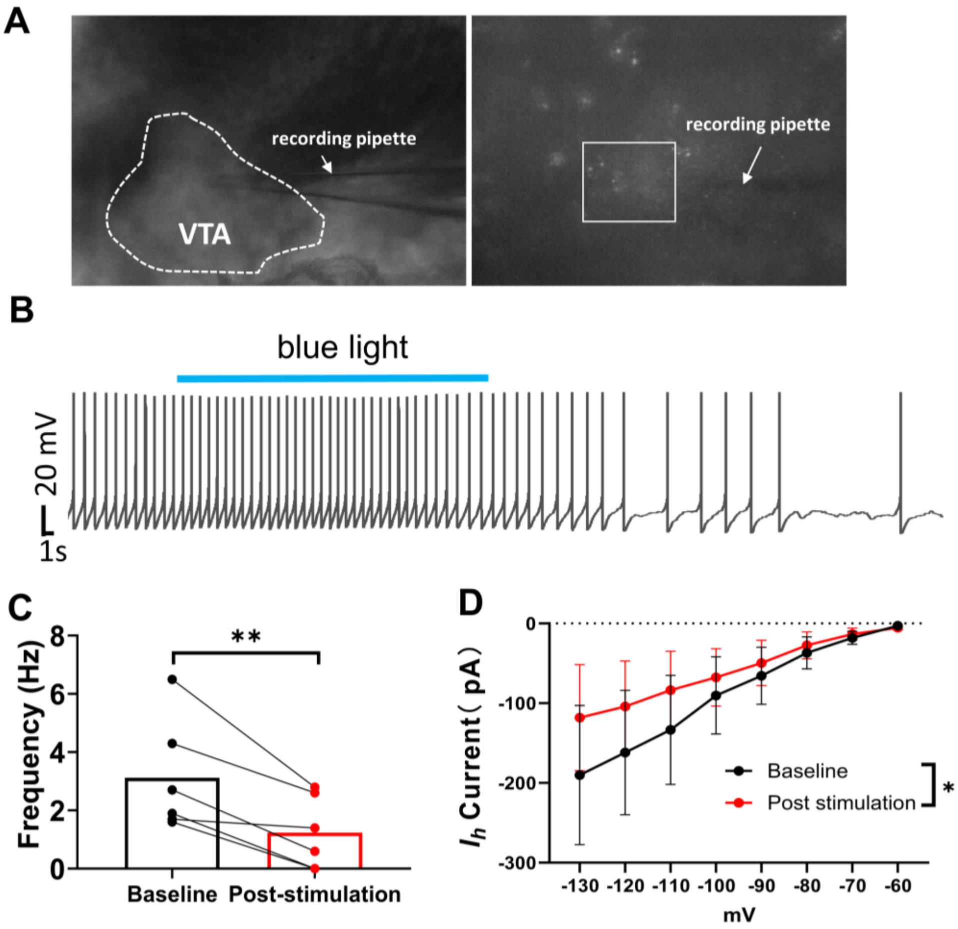

Supplementary Figure 8. Optogenetic activation of IL-13-expressing terminals in the VTA suppresses dopamine neuron activity and reduces I_h currents.

(A) Images of recording VTA neurons in acute brain slices from IL-13-Cre::Ai32 mice. The enlarged view of the recording area is shown in the right panel. The square box indicates the recording region enriched with ChR2-expressing terminals. Kynurenic acid (250 µM) and Bicuculine (10 µM) were added in the recording solution to block excitatory and inhibitory synaptic transmissions. (**B**) Representative trace showing the activity of a dopamine neuron before, during (20 s constant blue light), and after stimulation. (**C**) Summary data. **, $p<0.01$, paired t test. n=6 cells from 4 mice. (**D**) I_h currents at baseline and after optogenetic stimulation. $F (1, 5) = 7.57$, *, $p<0.05$, Two-way repeated measures ANOVA, for stimulation effect. Data are presented as mean± SEM.

References

1. Miao W, Zhao Y, Huang Y, Chen D, Luo C, Su W *et al.* IL-13 Ameliorates Neuroinflammation and Promotes Functional Recovery after Traumatic Brain Injury. *J Immunol* 2020; **204**(6)**:** 1486-1498.

2. Maher P, Conti B. Deciphering the pathways that protect from IL-13-mediated potentiation of oxidative stress-induced dopaminergic nerve cell death. *Cytokine* 2018; **103:** 114-120.

3. Mori S, Maher P, Conti B. Neuroimmunology of the Interleukins 13 and 4. *Brain Sci* 2016; **6**(2).

4. Morrison BE, Marcondes MC, Nomura DK, Sanchez-Alavez M, Sanchez-Gonzalez A, Saar I *et al.* Cutting edge: IL-13Ralpha1 expression in dopaminergic neurons contributes to their oxidative stress-mediated loss following chronic peripheral treatment with lipopolysaccharide. *J Immunol* 2012; **189**(12)**:** 5498-5502.

5. Xu X, Zheng S, Ren J, Li Z, Li J, Xu Z *et al.* Hypothalamic CRF neurons facilitate brain reward function. *Curr Biol* 2024; **34**(2)**:** 389-402 e385.
